# The impact of confinement on anxiety rates of the entourage of patients in the psychiatric hospital of Tunis

**DOI:** 10.1192/j.eurpsy.2021.1850

**Published:** 2021-08-13

**Authors:** H. Tanazefti, M. Beldi, A. Hajri, A. Maamri, H. Zalila

**Affiliations:** Faculty Of Medincine Of Tunis, RAZI HOSPITAL, TUNIS, Tunisia

**Keywords:** psychiatric patients’ accompanyings, confinement, Anxiety

## Abstract

**Introduction:**

The confinement did begot a recrudescence in the rate of stress within populations. Meanwhile, research targeting the mental health of the psychiatric patients’ accompanyings are scarce.

**Objectives:**

Detect and evaluate anxiety levels of patients’ entourage during confinement.

**Methods:**

It is a retrospective, descriptive and analytical study based on a random sample of People accompanying psychiatric patients from externals consultations service in the only Psychiatric Hospital in Tunisia. Data were been collected during the month of June 2020 via a 20 items questionnaire and a score HAD issued in Arabic.

**Results:**

One hundred thirty five accompanyings were surveyed. The age group was predominantly between 51 and 60 with a sex ratio of 0.31. Near half was the parents. eighty seven were unemployed, 38 stopped working due to confinement and 10 have been worked normally. A pathological anxiety HAD score (>7) was found in 36 accompanyings (26, 67 %). Amongst them, 19 had manifested symptoms. Anxiety levels are significantly much higher in accompanyings of patients with personality disorders (p=0.053). Otherwise, 52, 6% of accompanyings who stopped working felt more under pressure than before lockdown. In contrary to those who did not worked before at all (29.9 %) and those who continued working (10%).

**Conclusions:**

It seems that the entourage of mentally ill patients experience a continuous psychological distress, which was uncovered and marked in confinement period. Thus, it is necessary to establish screening programs, psychological education and early care to ensure their well-being.

**Disclosure:**

No significant relationships.

